# Biodegradation of Nodularin by a Microcystin-Degrading Bacterium: Performance, Degradation Pathway, and Potential Application

**DOI:** 10.3390/toxins13110813

**Published:** 2021-11-18

**Authors:** Mengxuan Yuan, Qin Ding, Rongli Sun, Juan Zhang, Lihong Yin, Yuepu Pu

**Affiliations:** Key Laboratory of Environmental Medicine Engineering, Ministry of Education of China, School of Public Health, Southeast University, Nanjing 210009, China; mxyuan@seu.edu.cn (M.Y.); dingqin@seu.edu.cn (Q.D.); sunrongli20609@163.com (R.S.); zhangjuan@seu.edu.cn (J.Z.); lhyin@seu.edu.cn (L.Y.)

**Keywords:** NOD, *Sphingopyxis*, biodegradation, degradation pathway, *mlr* gene cluster, recombinase

## Abstract

Currently, studies worldwide have comprehensively recognized the importance of *Sphingomonadaceae* bacteria and the *mlrCABD* gene cluster in microcystin (MC) degradation. However, knowledge about their degradation of nodularin (NOD) is still unclear. In this study, the degradation mechanism of NOD by *Sphingopyxis* sp. m6, an efficient MC degrader isolated from Lake Taihu, was investigated in several aspects, including degradation ability, degradation products, and potential application. The strain degraded NOD of 0.50 mg/L with a zero-order rate constant of 0.1656 mg/L/d and a half-life of 36 h. The average degradation rate of NOD was significantly influenced by the temperature, pH, and initial toxin concentrations. Moreover, four different biodegradation products, linear NOD, tetrapeptide H-Glu-Mdhb-MeAsp-Arg-OH, tripeptide H-Mdhb-MeAsp-Arg-OH, and dipeptide H-MeAsp-Arg-OH, were identified, of which the latter two are the first reported. Furthermore, the four *mlr* genes were upregulated during NOD degradation. The microcystinase MlrA encoded by the *mlrA* gene hydrolyzes the Arg-Adda bond to generate linear NOD as the first step of NOD biodegradation. Notably, recombinant MlrA showed higher degradation activity and stronger environmental adaptability than the wild strain, suggesting future applications in NOD pollution remediation. This research proposes a relatively complete NOD microbial degradation pathway, which lays a foundation for exploring the mechanisms of NOD degradation by MC-degrading bacteria.

## 1. Introduction

Cyanotoxins such as nodularin (NOD) and microcystin (MC) released by cyanobacterial cells pose a severe threat to the environment and human health, as evidenced by the destruction of aquatic food webs [1,2], mortality of marine mammals and birds [3], massive fish kills [4], and human diseases and even death [5]. NOD is predominantly produced by *Nodularia spumigena*, an ancient organism of the Baltic Sea that grows uncontrollably in the summer months annually [6]. In recent years, however, the occurrence of NOD and its detrimental effects have been frequently reported around the world, including Australia [7], Europe [8], New Zealand [9], Spain [10], and Greece [11]. Besides exerting tumor-promoting activity by specifically inhibiting protein phosphatases 1 and 2A [12], Ohta et al. demonstrated that NOD penetrates more easily into hepatocytes and is also a tumor initiator [13]. Cyclic pentapeptide NOD is composed of D-MeAsp, L-Arg, Adda, D-Glu, and Mdhb, coincidentally similar to heptapeptide MC in structure and biological activity [14,15,16].

Removing cyanotoxins from water has become a top priority owing to their significant hazards [17]. Indigenous microbial degradation is accepted as the most important route to eliminate cyanotoxins from natural water owing to its low capital cost, safety, high efficiency, and environmental friendliness [18,19]. Dozens of MC-degrading bacteria strains have been isolated from natural water since 1994, and most of them belong to the *Sphingomonadaceae* family [20,21,22]. Bourne et al. first reported that MC biodegradation by *Sphingomonas* sp. ACM 3962 is primarily dependent on the enzymatic pathway encoded by gene cluster *mlrBDAC* [23,24]. Subsequently, the homologous *mlr* gene cluster was also detected in other isolated *Sphingomonadaceae* bacteria, such as *Sphingopyxis* sp. C-1 [25], X20 [26], YF1 [27], and USTB-05 [28], *Sphingomonas* sp. MD-1 [29] and B9 [30], and *Novosphingobium* sp. THN-1 [31], with genera *Sphingopyxis* and *Sphingomonas* accounting for the majority. The *mlr* gene cluster encodes three hydrolysis enzymes and one oligopeptide transporter protein. The first enzyme encoded by the *mlrA* gene is responsible for opening the highly stable cyclic structure of MC by cleaving the Arg-Adda bond. The resulting linear product is then degraded to a tetrapeptide by the second enzyme encoded by the *mlrB* gene. The enzyme MlrC encoded by the *mlrC* gene hydrolyzes the tetrapeptide to Adda as the final nontoxic product and also exerts cleavage activity on the linear product to degrade it directly to Adda. The transporter protein encoded by the *mlrD* gene is supposed to transport MC and its degradation products into bacterial cells [23,24]. Currently, novel studies on fundamental knowledge about the biodegradation of strains of the *Sphingomonadaceae* family have focused on MC, while NOD has received little attention [19,21,32].

To the best of our knowledge, views regarding the presence of microorganisms capable of degrading NOD in natural water date back to the 1990s [33]; however, the first native strain was not isolated and characterized until 2005 [34]. Until now, we have only found a few strains capable of degrading NOD [35,36], and few studies about the pathways, genes, and enzymes involved in NOD biodegradation have been conducted. Considering that NOD and MC are the most toxic and widely distributed, with similar structure and amino acid composition, the application of *Sphingomonadaceae* strains capable of degrading MC and/or NOD could facilitate understanding of biological attenuation for cyanotoxins, although this fact is rarely noticed. Therefore, there are gaps regarding whether it can degrade NOD, whether it shares a set of degradation genes with MC or mobilizes an entirely new degradation mechanism, and whether it has potential value in practical applications.

We recently found that *Sphingopyxis* sp. m6, an indigenous strain isolated from Taihu Lake with degradation activity against MC [37], was capable of degrading NOD. We evaluated the NOD degrading activity of this bacterium under different culturing environments. The possible pathway associated with NOD degradation was inferred from the discovery of several degradation products during the degradation process. Concomitantly, the expression dynamics of the MC degradation gene cluster carried by strain m6 were tracked throughout the degradation experiment. The correlation between the expression of the four *mlr* genes and NOD degradation rates was demonstrated. Finally, it was verified that MlrA encoded by the *mlrA* gene played an important hydrolytic role in NOD biodegradation, and the degradation efficiency and environmental adaptability of recombinant MlrA were investigated to preliminarily explore its application potential.

## 2. Results

### 2.1. Biodegradation Kinetics of NOD by Sphingopyxis sp. m6

**Figure 1:**
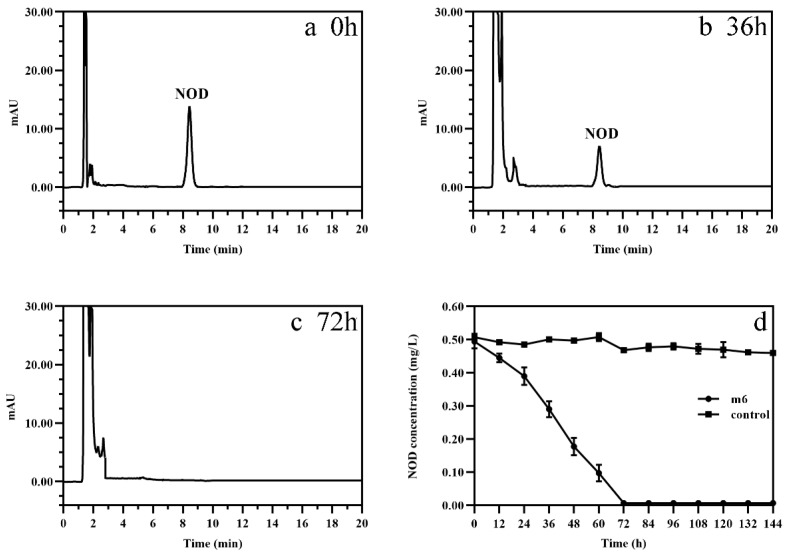
HPLC profiles for the microbial degradation of NOD by strain m6 at (**a**) 0 h, (**b**) 36 h, and (**c**) 72 h. (**d**) Biodegradation curve of NOD by strain m6. The error bars represent the standard deviation (*n* = 3).

After incubation of strain m6 with 0.50 mg/L NOD, the peak area value of NOD monitored by high performance liquid chromatography (HPLC) decreased over time and was almost zero at 72 h, which meant that the NOD concentration was reduced below the detection threshold for HPLC (Figure 1a–c). In contrast, almost no reduction in NOD was observed in the bacteria-free group, excluding physical, chemical, and other abiotic degradation. Figure S1 presents the standard curve of NOD quantified by HPLC.

The biodegradation kinetics of NOD showed that strain m6 decomposed NOD immediately without any lag period. The degradation rate initially increased and then decreased, reaching a maximum of 0.23 mg/L/d at 48 h, with an average degradation rate of 0.16 mg/L/d (Figure 1d). The kinetics model was set up based on the analysis of experimental dimensionless concentrations over the degradation time. Compared with first- or second-order reaction kinetics, the regression coefficient *R*^2^ value suggested that the zero-order reaction kinetics is better for degradation of NOD (*R*^2^ = 0.987). The rate constant for NOD biodegradation was computed utilizing the following equation:

(1) $$c_{0}-c=kt$$

where k is the rate constant, $c_{0}$ and c are the initial concentration of NOD and the NOD concentration at time *t*, respectively. The degradation rate constant of NOD was calculated to be 0.1656 mg/L/d. The half-life (*t*_1/2_) of zero-order reaction kinetics can be estimated as follows:

(2) $$t_{1/2}=\frac{c_{0}}{2k}$$

Therefore, the *t*_1/2_ is approximately 36 h.

### 2.2. Factors Affecting Microbial Degradation of NOD

**Figure 2:**
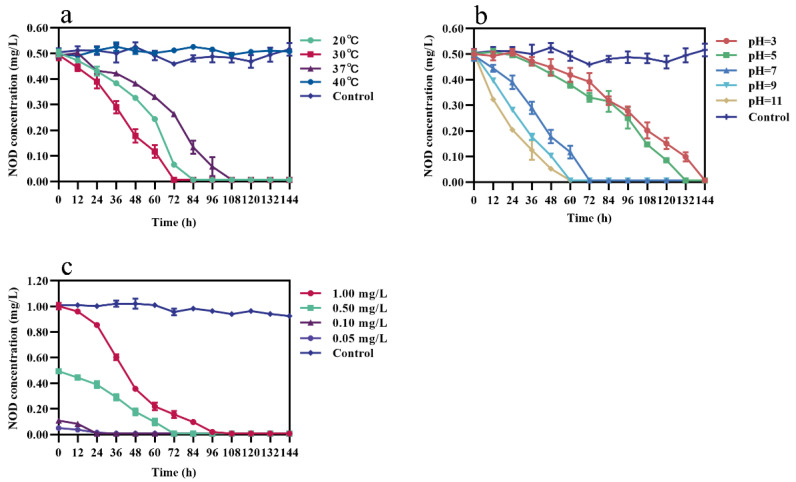
Effect of different incubation conditions on the NOD-degrading rate of stain m6. (**a**) Incubation temperatures (pH = 7, 0.50 mg/L NOD), (**b**) pH (30 °C, 0.50 mg/L NOD), and (**c**) NOD concentrations (30 °C, pH = 7). The error bars represent the standard deviation from triplicate experiments.

Single-factor experiments were performed under different incubation conditions. The results showed that the average degradation rate of NOD is strongly influenced by incubation temperatures, pH, and initial NOD concentrations. Under pH 7 and 0.50 mg/L NOD, bacterium m6 degraded NOD with an average rate of 0.14, 0.16, and 0.11 mg/L/d at 20, 30, and 37 °C, respectively (Figure 2a). The rate constant increased from 0.1344 mg/L/d to 0.1656 mg/L/d as the temperature rises from 20 °C to 30 °C. However, it decreased to 0.1152 mg/L/d when the temperature reached 37 °C. The optimal reaction temperature was 30 °C, whereas the toxin concentration barely decreased in the control and 40 °C groups. Figure 2b illustrated that at 30 °C, 0.50 mg/L NOD was removed, with the average rate of 0.08, 0.09, 0.16, 0.20, and 0.20 mg/L/d at pH 3, 5, 7, 9, and 11, respectively. The degradation of NOD was sensitive to the initial pH of the reaction system, with rate constants (0.1920–0.1928 mg/L/d) observed at pH 9 and 11 more than twice as high as under acidic environments. At 30 °C and pH 7, different initial concentrations of 1.00, 0.50, 0.10, 0.05 mg/L NOD were degraded at the rate of 0.22, 0.16, 0.10, and 0.03 mg/L/d, respectively (Figure 2c). Collectively, there was a lag period before degradation started only at pH 3 and 5. Strain m6 exhibited the highest degradation rate (0.22 mg/L/d) of NOD at 30 °C and pH 7 with toxin concentration of 1.00 mg/L.

### 2.3. Identification of NOD Biodegradation Products

The total ion chromatogram (TIC) showed five substance peaks at different stages of degradation by strain m6 (Figure S2). The ten predominant fragment ions proved that the compound with *m/z* 825.4494 appearing at 8.359 min in TIC was NOD (Figures S2a and S3a,b; Table 1), as all amino acids were present. The MS spectrum of TIC at 7.836 min exhibited a major ion at *m/z* 692.3619 and a protonated molecular ion at *m/z* 843.4600 (Figures S2b and S3c). Based on all ions and bonds found in the fragmentation spectrum, except for the Arg-Adda bond, *m/z* 843.4600 with 18 Da more than the mass value of NOD was identified as hydrolyzed NOD [M + H_2_O + H]^+^ (Figure S3c,d; Table 1). The major ion at *m/z* 692.3619 was obtained by losing the amino NH_2_ group and the PhCH_2_CHOCH_3_ group (*m/z* 135.0805) from the linear product through random breakage. In the samples incubated with strain m6, two ions were present at 4.934 min of TIC (Figure S2d,e). Eight main fragment ions, 70.0646 [C_4_H_8_N]^+^, 158.0912 [Arg + OH − NH]^+^, 175.1186 [Arg-OH + 2H]^+^, 183.1146 [Mdhb-MeAsp + H − COOH]^+^, 201.0960 [Arg + OH + CO]^+^, 258.1558 [MeAsp-Arg + OH − COOH]^+^, 357.2228 [Mdhb-MeAsp-Arg + H − COOH]^+^, 401.2125 [M + OH + 2H]^+^ (Figure S4d; Table 1), provide evidence that one with *m/z* 401.2137 is a tripeptide H-Mdhb-MeAsp-Arg-OH (Figure S4c). The other molecular ion with *m/z* 304.1615 was fragmented into several ions, including *m/z* 70.0652 [C_3_H_4_NO]^+^, 158.0912 [Arg + OH − NH]^+^, 175.1195 [Arg-OH + 2H]^+^, and 304.1664 [M + OH + 2H]^+^ (Figure S5b; Table 1), demonstrating that this compound contained only MeAsp and Arg (Figure S5a). The last substance peak with *m/z* 530.2565 appeared in TIC at 4.789 min (Figures S2c and Figure S4a), revealing ten fragmentation products: 70.0655 [C_4_H_8_N]^+^, 158.0932 [Arg + OH − NH]^+^, 175.1186 [Arg-OH + 2H]^+^, 209.0922 [Glu-Mdhb + H − OH]^+^/[Mdhb-MeAsp + H − OH]^+^, 227.1009 [Glu-Mdhb + H]^+^/[Mdhb-MeAsp + H]^+^, 304.1632 [MeAsp-Arg-OH + 2H]^+^, 401.2285 [Mdhb-MeAsp-Arg-OH + 2H]^+^, 494.2389 [M + H − OH]^+^, and 512.2464 [M + H]^+^ (Figure S4b; Table 1), confirming that the substance was a tetrapeptide H-Glu-Mdhb-MeAsp-Arg-OH. No product was detected in the standard NOD samples.

The dynamics of the peak intensity for NOD and all degradation products detected by mass spectrometry are displayed in Figure S6. With the removal of NOD, degradation products progressively appeared. The signal intensity of linear NOD increased rapidly to a maximum at 72 h (Figure S6b), while the others required 108 h (Figure S6c–e). All products exhibited a downward trend after reaching the peak. Only the intensity value of the linear NOD dropped to almost zero (Figure S6b).

### 2.4. Expression of mlr Gene Cluster during NOD Biodegradation

**Figure 3:**
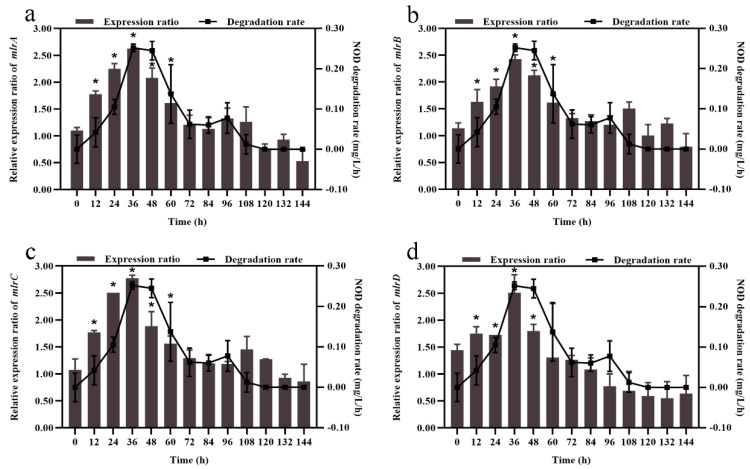
Kinetic relationships between NOD degradation rate and *mlr* gene cluster expression. (**a**) *mlrA* gene, (**b**) *mlrB* gene, (**c**) *mlrC* gene, and (**d**) *mlrD* gene. Significant differences are indicated in comparison with the control by *t*-test. *, *p* < 0.05. The error bars represent the standard deviation of means for triplicate.

After exposure to NOD, the expression dynamics of the four *mlr* genes in bacterium m6 were similar (Figure 3). Specifically, during the 144-h experimental period, the relative expression ratios of the *mlr* gene cluster experienced a rough three-step process of gradual increase, reaching the highest at 36 h and then decreasing to below the control level (Figure 3). At 36 h, the relative expression ratio of the *mlrC* gene was the largest (2.8-fold upregulation) (Figure 3c), while the *mlrB* gene had the minimum 2.4-fold upregulation (Figure 3b). After the peak, the expression level of the *mlrD* gene decreased most rapidly (Figure 3d). Figure 3 attempts to explain the kinetic relationships between the NOD degradation rate and the relative expression ratio of the four genes. For each, the rate of NOD degradation follows its expression trend, simultaneously reaching its maximum (Figure 3). There was a positive correlation between NOD degradation rate and the gene expression ratio of *mlrA* (*R*^2^ = 0.714, *p* < 0.01), *mlrB* (*R*^2^ = 0.769, *p* < 0.01), *mlrC* (*R*^2^ = 0.5723, *p* < 0.01), and *mlrD* (*R*^2^ = 0.5972, *p* < 0.01), respectively.

### 2.5. NOD Degradation and Its Product by Recombinant MlrA

**Figure 4:**
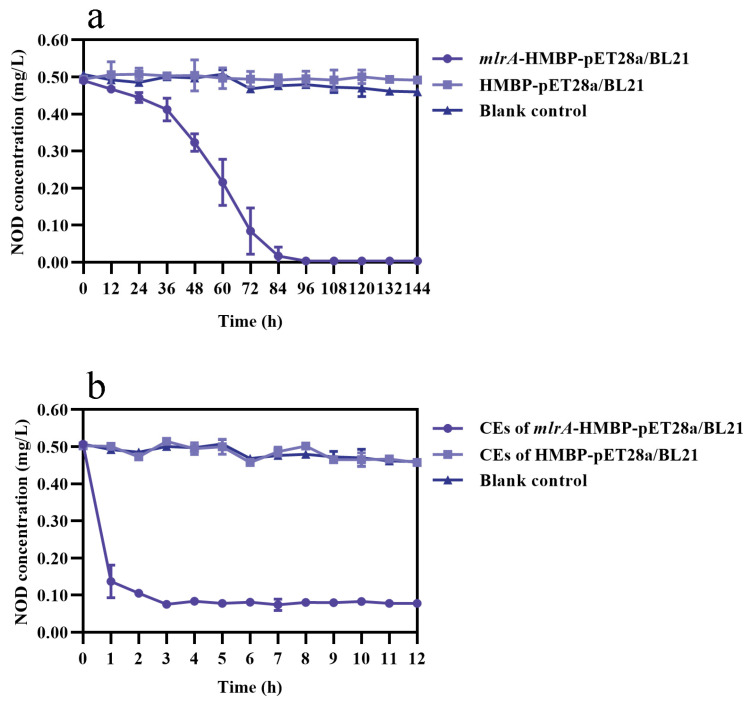
NOD degradation curves of (**a**) intact recombinant *mlrA*-HMBP-pET28a/BL21 and (**b**) CEs from the recombinant. Bars represent the standard deviation of the means for triplicate.

NOD concentration was obviously decreased in both recombinant *mlrA*-HMBP-pET28a/BL21 and cell-free extracts (CEs) of the recombinant (Figure 4) while remaining essentially unchanged in the corresponding negative controls, suggesting that the *mlrA* gene-encoded enzyme (MlrA) may cause the loss of NOD. The absence of changes in the blank controls also ruled out self-catabolism. Nevertheless, divergent degradation rates of NOD were observed between intact recombinant cells and the CEs of the recombinant cells. A non-negligible phenomenon was that CEs from the recombinant sharply degraded more than 70% NOD within the first hour at a rate of 0.37 mg/L/h (Figure 4b). In contrast, intact cells only degraded 5% NOD in the first hour, which was significantly lower than its CEs at the same time point (*p* < 0.01). The average rate of intact recombinant was 0.12 mg/L/d (Figure 4a). Consequently, this series of tests indicated that recombinant MlrA readily degraded the majority of NOD in a short period of time, showing higher NOD removal efficiency than the wild strain and recombinant.

The MS spectrum exhibited two major ion peaks at *m/z* 825.4562 and 843.4596. The monitored intensity of *m/z* 843.4596 consistently enhanced and showed no signs of attenuation, and its fragment ions were essentially identical to those previously described in Section 2.3. Hence, it was confirmed that MlrA is responsible for the first step in NOD bio-catalysis by strain m6, opening the Arg-Adda bond in the ring-like structure.

### 2.6. Environmental Adaptability Assay of Recombinant MlrA

**Figure 5:**
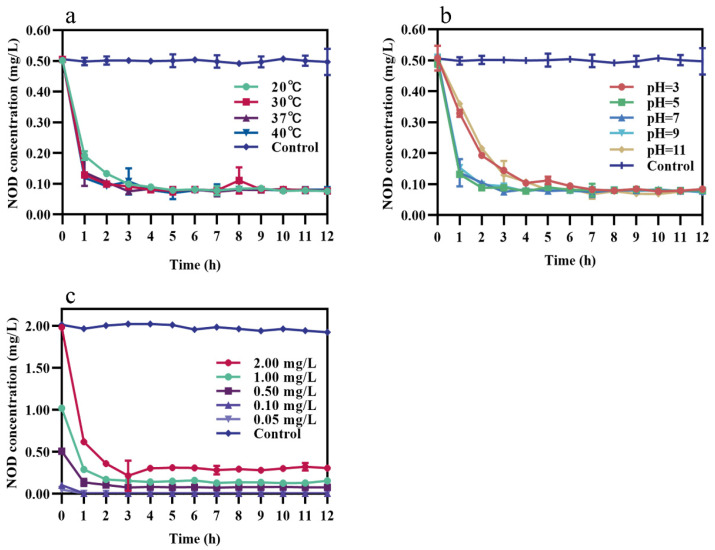
Effect of different conditions on the NOD-degrading rate of recombinant MlrA. (**a**) Temperatures (pH = 7, 0.50 mg/L NOD), (**b**) pH (37 °C, 0.50 mg/L NOD), and (**c**) NOD initial concentrations (37 °C, pH = 7). The error bars represent the standard deviation from triplicate experiments.

As shown in Figure 5a, recombinant MlrA showed higher activity at 30, 37, and 40 °C, degrading more than 70% NOD in the first hour but 60% at 20 °C. However, there was no statistically significant difference in the degradation rates of recombinant MlrA (*p* > 0.05). Proteolytic activity was lower under solid acid (pH = 3) and strongly alkaline (pH = 11) conditions, with only approximately 30% NOD being degraded in the first hour at pH 3 compared to less than 30% at pH 11 (Figure 5b). The degradation curves for recombinant MlrA at pH 5, 7, and 9 largely overlapped (Figure 5b). Interestingly, no statistically significant differences in degradation rates were found between the temperature and pH groups (*p* > 0.05). In addition, enzyme activity was affected by the initial NOD concentration. The degradation rate in the first hour was proportional to the initial toxin concentration (Figure 5c). These features enhance the application potential of recombinant MlrA.

## 3. Discussion

Globally, the frequency, intensity, and duration of harmful cyanobacterial blooms (HCBs) are increasing due to the eutrophication of water bodies [38,39]. Furthermore, cyanotoxins produced by HCBs have potent carcinogenic and even lethal effects on public health [40,41]. Among the numerous methods for removing cyanotoxins from natural water, biodegradation is the primary mechanism for eliminating toxins and is considered an eco-friendly and environmentally benign technology [42,43]. Studies on specific mechanisms for the degradation gene cluster *mlr* carried by *Sphingomonadaceae* bacteria, which account for a large proportion of MC-degrading bacteria, have advanced our understanding of the biological attenuation of MC in nature [20,21,44]. Nevertheless, the field of its bio-catalytic degradation mechanisms for NOD still needs to be addressed.

In the present study, *Sphingopyxis* sp. m6, an efficient MC degrader isolated from Lake Taihu [37], was also able to degrade NOD (Figure 1). Not all MC-degrading strains can decompose NOD, and only a few strains have been reported prior to this study; however, no further elaboration was given [34,35,45,46]. The lag phase is a significant factor affecting biodegradation kinetics [32]. Under average temperature and neutral environment, most reported functional bacteria have a lag period ranging from several hours to a few days before commencing degradation [32,47,48,49]. In contrast, for strain m6, there was no significant lag period for the degradation of either NOD or MC-LR at 30 °C and pH 7 (Figure 1d) [37]. This may be because strain m6 is an indigenous microorganism isolated from Lake Taihu, which has been chronically contaminated with blooms [50]. As indicated in some previous studies, the lag period can be shortened, and in some circumstances even eliminated, by pre-exposure or frequent exposure to cyanotoxins [51,52]. Therefore, the literature that integrates the history of algal toxin exposure into factors of biodegradation rate is rational [53]. The diversity of NOD concentrations used, reaction conditions, culture density of the studied strain, and the methods employed resulted in no comparable degradation rates between strain m6 and other previously reported bacteria [35,36,45]. However, at the same concentration, strain m6 did not respond consistently to NOD and MC-LR. This could be attributed to the higher relative specificity of the degradative enzymes for MC-LR, that is to say, the binding sites of the enzymes are more compatible with MC-LR.

Environmental conditions such as temperature or pH are related to cyanobacterial blooms and affect the biodegradation process of cyanobacterial toxins [16]. Many studies have shown that optimal biodegradation occurs at approximately 30 °C under neutral or weakly alkaline conditions [54,55,56]. Strain m6 showed the best degradation efficiency at 30 °C, but almost completely lost its degradation ability at 40 °C (Figure 2a). One reason for this is that higher temperatures have an adverse effect on the survival of organisms, although the probability of collisions between particles increases. The pH of natural water is usually above 8 during cyanobacterial blooms because of enhanced photosynthesis due to the consumption of carbon dioxide [57,58]. Interestingly, the average degradation rate of NOD by strain m6 was more rapid with an increase in pH, a phenomenon that is quite the opposite of its degradation of MC-LR (Figure 2b) [37]. A possible explanation could be that some sequences more adapted to high pH for regulating protease activity are mobilized only during the NOD degradation. Detailed studies are required to test this hypothesis. It is worth noting that under acidic conditions there was a lag period before strain m6 started to degrade NOD (Figure 2b), primarily because the synthesis and activation of degrading enzymes may be inhibited to some extent under unsuitable conditions. In addition, the average degradation rate correlated positively with the initial NOD concentration (Figure 2c), which is consistent with the results of previous studies [59,60].

**Figure 6:**
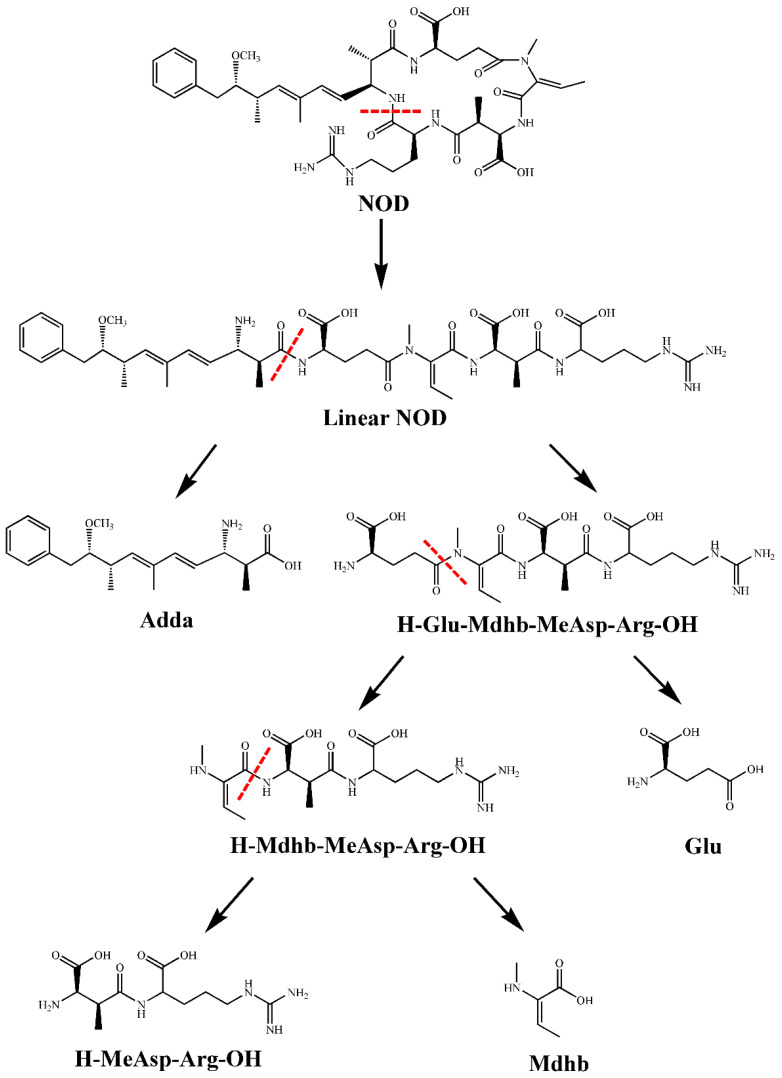
Proposed biodegradation pathway of NOD by strain m6.

The first study focusing on NOD biodegradation products was performed using *Sphingomonas* sp. B-9 cells. It found a degradation intermediate linear NOD and a final product Adda [45,61], similar to the degradation route discovered later by *Sphingopyxis* sp. USTB-05 [46]. In addition to two (linear NOD and Adda) belonging to previously reported compounds, Mazur-Marzec et al. found three novel degradation products in their study of NOD degradation by natural microbial communities in sediments: H-Glu-Mdhb-MeAsp-Arg-OH, H-Adda-Glu-Mdhb-MeAsp-OH, and H-Adda-Glu-Mdhb-MeAsp-Cit-OH [62]. Four degradation products were found in our study, including two new products: H-Mdhb-MeAsp-Arg-OH and H-MeAsp-Arg-OH (Figures S4c and S5a). The non-hydrolytic degradation pathways proposed by Edwards et al., such as demethylation of Mdhb, decarboxylation of MeAsp, and modification of Adda, were not observed during the degradation process in this study [63]. Figure 6 describes the specific degradation pathway of NOD by *Sphingopyxis* sp. m6. First, linear NOD was generated by breaking the Arg-Adda bond to open the cyclic pentapeptide. Next, the Adda-Glu bond was hydrolyzed to produce a tetrapeptide (H-Glu-Mdhb-MeAsp-Arg-OH). The tetrapeptide was further decomposed to a tripeptide (H-Mdhb-MeAsp-Arg-OH) by cleavage of the peptide bond at Glu-Mdhb. Finally, strain m6 successfully recognized and hydrolyzed the Mdhb-MeAsp bond to form a dipeptide (H-MeAsp-Arg-OH). These novel findings provided valuable supplements to previous studies on single strain and proposed a relatively complete NOD biodegradation pathway. Surprisingly, the entire degradation process did not reveal the presence of the traditional final product Adda and dimeric Adda that often occurs during NOD biodegradation. It should be noted that strain m6 has a metabolic pathway that degrades Adda so quickly that the signal intensity is below the detection threshold. Further degradation of Adda as a degradation intermediate has been reported [27,37].

Case studies of different *Sphingomonadaceae* strains have led to a consensus that the *mlr* gene cluster plays an essential role in the degradation of MC and its variants [20,21,44]. However, less information was available on NOD, even though it has a vital significance for understanding the environmental attenuation of NOD. In this study, detection of NOD biodegradation products, including linear NOD, implied that MlrA encoded by the *mlrA* gene acts similarly on NOD and MC by cleaving their ring structure at the Adda-Arg peptide bond. The results of the genetic analysis showed that exposure to NOD upregulates the expression of four *mlr* genes (Figure 3). The trend of *mlr* gene expression mostly coincided with that reported in other studies on MC degradation mechanisms, implying that this gene cluster is also involved in NOD degradation [37,64,65]. The positive correlation between NOD degradation rate and *mlr* gene cluster expression provides evidence to support a contention that biodegradation kinetics are regulated by the expression of this gene cluster, and the more considerable *R*^2^ value between *mlrA* gene expression and degradation rate indicates the importance of the *mlrA* gene in NOD biodegradation by strain m6.

This study further validated the role of the *mlrA* gene with the help of intact recombinant *mlrA*-HMBP-pET28a/BL21 and its CEs. The results definitively confirm that MlrA generates linear NOD as the first product by hydrolyzing the Arg-Adda bond in NOD. Combined with the previous study by Ding et al., it was suggested that the target substrates of MlrA in strain m6 are not limited to MC, which is consistent with the case of *Sphingopyxis* sp. USTB-05 [66]. It is noteworthy that this finding cannot be directly applied to all strains because the amino acid sequencing and structure of the specific protease encoded by the *mlrA* gene may differ in various bacteria. Bourne et al. found that MlrC recognizes and hydrolyzes the Adda-Glu bond of MC [24]. This finding was subsequently confirmed in many MC-degrading bacteria containing the *mlr* gene cluster [21,44]. Based on the fact that the Adda-Glu bond is also present in NOD and the upregulation expression of the *mlrC* gene during degradation, it is speculated that the *mlrC* gene regulates the hydrolysis reaction in the second step of NOD degradation pathway. However, the specific function of the *mlrB* gene needs further investigation due to the structural differences between NOD and MC. Degradation of intact recombinant indicated that recombinant MlrA does not exist in the host as an inclusion body (Figure 4a) [67]. Compared with the relatively moderate degradation ability of strain m6, the biodegradation curve of recombinant MlrA dropped sharply in the first hour (Figure 4b) and tended to be horizontal after 3 h, with trace amounts of toxin remaining even at 48 h (data not shown). This may be a consequence of the presence of other complex components in recombinant CEs preventing recombinant MlrA from binding to the substrate and performing catalysis when the NOD concentration decreases to a low level in a confined space [68]. Although the concentration of recombinant MlrA from CEs and the density of strain m6 could not be compared, it was concluded that recombinant MlrA could be activated within an extremely short period of time to initiate rapid degradation. The environmental adaptability of recombinant MlrA is discussed in this paper. Recombinant MlrA exhibited high catalytic activity even at high temperatures and extreme pH values (Figure 5). This finding paves the way for the subsequent introduction of recombinant enzymes into environmental applications, as corroborated by several studies on the potential applications of recombinant MlrA directly as a sustainable bioremediation product [68,69,70,71].

## 4. Conclusions

In this study, an indigenous bacterium, *Sphingopyxis* sp. m6, with strong MC degradation capability was able to degrade NOD. The biodegradation of NOD by strain m6 followed zero-order reaction kinetics. The average degradation rate of NOD was affected by the temperature, pH, and initial NOD concentrations. The MS analysis identified four products, of which two (H-Mdhb-MeAsp-Arg-OH and H-MeAsp-Arg-OH) have never been reported before. A relatively complete degradation pathway was proposed in this study; however, the Adda or Adda dimer was not found. The *mlr* gene cluster was highly transcribed during the catabolism of NOD by strain m6. It was further confirmed that MlrA encoded by the *mlrA* gene is responsible for the first step of the NOD biodegradation pathway by recognizing and hydrolyzing the Arg-Adda bond. Moreover, heterologous expression of recombinant MlrA significantly improved the biodegradation efficiency of NOD. The broad adaptability of recombinant MlrA to the environment provides preliminary evidence of its application potential.

## 5. Materials and Methods

### 5.1. Bacteria and Reagents

The previously obtained strains *Sphingopyxis* sp. m6, recombinant *mlrA*-HMBP-pET28a/BL21, and HMBP-pET28a/BL21 were investigated in this study [37]. Standard NOD (C_41_H_60_N_8_O_10_, ≥95% purity) purchased from APExBIO (Houston, USA) was dissolved in methanol at a concentration of 100 mg/L and stored at −20 °C. The mineral salt medium (MSM) was used as an essential component of the experimental systems. The real-time quantitative polymerase chain reaction (RT-qPCR) kit was supplied by TaKaRa (Kusatsu, Japan). Trifluoroacetic acid (Sinopharm Chemical Reagent Co., Ltd. Shanghai, China) and HPLC-grade methanol (TEDIA Co., Fairfield, OH, USA) were applied to the liquid phase. UPLC grade methanol (Merck KGaA, Darmstadt, Germany) and formic acid (Fisher Scientific, Shanghai, China) were used for mass spectrometry. 

### 5.2. NOD Degradation Capability of Strain m6

Strain m6 was incubated in Luria-Bertani (LB) medium at 30 °C with shaking at 150 rpm, harvested using centrifugation (5000× *g*, 10 min, and 4 °C) during the logarithmic growth period (OD600 = 0.60 ± 0.05), washed three times, and then resuspended in 3 mL of sterile MSM containing 0.50 mg/L NOD. A certain amount (100 µL) of each sample was withdrawn every 12 h for six days. After centrifugation (12,000× *g*, 15 min, and 4 °C), the residual concentrations of NOD in the supernatants were measured using HPLC (Agilent 1260, Santa Clara, CA, USA). A bacteria-free group was used as a control.

### 5.3. NOD Degradation under Different Environmental Conditions

Three series of experiments were designed to simulate various environmental conditions. First, four different temperatures (20, 30, 37, and 40 °C) were tested with 0.50 mg/L NOD at pH 7. Second, five different pH values (pH = 3, 5, 7, 9, and 11) were executed with 0.50 mg/L NOD at 30 °C. Finally, four different initial NOD concentrations (0.05, 0.10, 0.50, and 1.00 mg/L) were conducted with pH 7 at 30 °C. The control groups contained 0.50 or 1.00 mg/L NOD without bacteria cells and were cultured at 30 °C and pH 7. Samples of 100 µL were taken every 12 h for six days and the concentrations of remaining NOD in supernatants were then detected by HPLC.

### 5.4. Quantification of NOD by HPLC

NOD was quantified by HPLC using a Zorbax Extend C18 column (2.1 × 50 nm, 1.8 µm, Agilent, Santa Clara, CA, USA) with a variable wavelength detector (VWD) at 238 nm. The mobile phase consisted of HPLC-grade methanol (B phase) and Milli-Q water containing 0.05% trifluoroacetic acid (A phase) (53:47, *v*/*v*) was set for chromatographic separation at a flow rate of 1.00 mL/min. The column temperature was maintained at 40 °C throughout the entire process, and the injection volume was 20 µL. 

### 5.5. Identification of Degradation Products

Ultra-performance liquid chromatography-tandem mass spectrometry (UPLC-MS/MS) (triple TOF 5600+, AB Sciex, Redwood, CA, USA) was used to identify NOD and its degradation products. First, 5 µL of Supernatant was injected into UPLC with an Acquity UPLC HSS T3 column (2.1 × 100 mm, 1.8 µm, Waters, Milford MA, USA). The mobile phase was a mixture of UPLC grade methanol (B phase) and Milli-Q water containing 0.1% formic acid (A phase). The percentage of B phase was started at 2% for 2 min, ramped linearly to 98% over 12 min, maintained at 98% from 14 min to 17 min, and then immediately dropped to 2% and held for 3 min at a constant flow rate of 0.30 mL/min for a total run time of 20 min. The MS system connected to the UPLC was equipped with a tandem quadrupole and atmospheric pressure chemical iosziaa-lion (APCI) source. The conditions were as follows: positive ionization mode, full scanning from *m/z* 100–1200, ionspray voltage floating of 5000 V, declustering potential of 80 V, and collision energy of 35 V.

### 5.6. Detection of mlr Gene Cluster Expression

To study whether degradation of NOD by strain m6 shares a standard set of degradation mechanisms with MC-LR, the five specific primers, q*mlrA*F/q*mlrA*R, q*mlrB*F/q*mlrB*R, q*mlrC*F/q*mlrC*R, q*mlrD*F/q*mlrD*R, and q16SF/q16SR, were designed based on the sequencing results of Ding et al. [37]. Total RNA from strain m6 in the degradation system was extracted with TRIzol (Life Technologies, Carlsbad, CA, USA) every 12 h until NOD disappeared entirely and was then reverse-transcribed to cDNA using a Reverse Transcription Kit (Takara Bio, Shiga, Japan). RT-qPCR was performed using SYBR Green Real-time PCR Master Mix (Toyobo, Osaka, Japan). The NOD-free control group was prepared in the same manner. The relative expression ratio was calculated by 2^−ΔΔCt^ method, and the 16S gene was used as the reference gene [72].

### 5.7. NOD Degradation by Intact Recombinant and CEs of the Recombinant

The *mlrA* gene heterologous expression recombinant (*mlrA*-HMBP-pET28a/BL21) and control strain (HMBP-pET28a/BL21), which were previously constructed in our laboratory (specific information will be published in another article), were independently inoculated in LB medium containing 50 µg/mL kanamycin and incubated at 37 °C with a shaking rate of 200 rpm/min until OD_600_ reached 0.60 ± 0.05. Isopropyl-β-D-thiogalactopyranoside (IPTG) at a final concentration of 0.50 mM was added in order to induce the expression of MlrA. After 4 h, the organisms were collected by centrifugation at 5000× *g* for 10 min at 4 °C. The bacteria were washed three times with Milli-Q water before being resuspended in 1 mL Milli-Q water. The suspensions were then divided into two aliquots. One aliquot was used to test the NOD degradation capability of intact cells, and the other aliquot was sonicated in a 4 °C water bath for 20 min, alternating every working time of 3 s with 3 s intervals during this period to prevent high temperature from inactivating the target enzyme. The ultrasonic-treated suspension was centrifuged at 4 °C and 12,000× *g* for 30 min to discard debris, and the supernatants were collected as CEs of recombinant *mlrA*-HMBP-pET28a/BL21.

To test the NOD degradation activity of recombinant MlrA, the suspensions of intact recombinant and CEs of the recombinant were spiked into sterile water with an initial concentration of 0.50 mg/L NOD, respectively. The suspensions of HMBP-pET28a/BL21 and CEs from HMBP-pET28a/BL21 were used as negative controls. A reaction system containing only NOD was used as the blank control. All tubes were incubated at 37 °C and shaken at 200 rpm. Samples (100 µL) were removed periodically, and methanol (two-fold volume) was added to terminate the enzyme reaction. After centrifugation at 4 °C and 15,000× *g* for 15 min, the residual NOD concentration and degradation products in the supernatants were detected by HPLC and UPLC-MS/MS.

### 5.8. Activity Assay of Recombinant MlrA

To evaluate the application potential of recombinant MlrA, CEs from the recombinant were studied to test degradability at different temperatures (20, 30, 37, and 40 °C), pH (3, 5, 7, 9, and 11), and initial NOD concentrations (0.05, 0.10, 0.50, 1.00, and 2.00 mg/L). CEs from HMBP-pET28a/BL21 cells were used as controls.

## Figures and Tables

**Table 1:** Fragmentation ions of NOD and its biodegradation products.

Detected Substance	Observed Fragmentions *m/z*	Predicted Fragment Structure
NOD	135.0799	PhCH_2_CHOCH_3_
	163.1105	[PhCH_2_CHOCH_3_CHCH_3_]^+^
	227.1032	[Glu-Mdhb + H]^+^/[Mdhb-MeAsp + H]^+^
	366.1738	[Mdhb-MeAsp-Arg + H − NH_3_]^+^
	389.2084	[C_11_H_15_O-Glu-Mdhb + H]^+^
	674.3511	[M + 2H − 135 − NH_3_]^+^
	691.3797	[M + 2H − 135]^+^
	781.4617	[M + H − CO_2_]^+^
	808.4258	[M + 2H − NH_3_]^+^
Linear NOD (H-Glu-Mdhb-MeAsp-Arg-Adda-OH)	135.0775	PhCH_2_CHOCH_3_
	175.1210	[Arg-OH + 2H]^+^
	304.1605	[MeAsp-Arg-OH + 2H]^+^
	556.2303	[CO-Glu-Mdhb-MeAsp-Arg-OH]^+^
	586.2816	[CH_3_CH_2_CHO-Glu-Mdhb-MeAsp-Arg-OH + 2H]^+^
	692.3611	[M + H_2_O − 151 + H]^+^
	826.4355	[M + H − NH_3_]^+^
Tetrapeptide (H-Glu-Mdhb-MeAsp-Arg-OH)	70.0655	[C_4_H_8_N]^+^
	158.0932	[Arg + OH − NH]^+^
	175.1186	[Arg-OH + 2H]^+^
	209.0922	[Glu-Mdhb + H − OH]^+^/[Mdhb-MeAsp + H − OH]^+^
	227.1009	[Glu-Mdhb + H]^+^/[Mdhb-MeAsp + H]^+^
	304.1632	[MeAsp-Arg-OH + 2H]^+^
	401.2285	[Mdhb-MeAsp-Arg-OH + 2H]^+^
	494.2389	[M + H − OH]^+^
	512.2464	[M + H]^+^
Tripeptide (H-Mdhb-MeAsp-Arg-OH)	70.0646	[C_4_H_8_N]^+^
	158.0912	[Arg + OH − NH]^+^
	175.1186	[Arg-OH + 2H]^+^
	183.1146	[Mdhb-MeAsp + H − COOH]^+^
	201.0960	[Arg + OH + CO]^+^
	258.1558	[MeAsp-Arg + OH − COOH]^+^
	357.2228	[Mdhb-MeAsp-Arg + H − COOH]^+^
	401.2125	[M + OH + 2H]^+^
Dipeptide (H-MeAsp-Arg-OH)	70.0652	[C_3_H_4_NO]^+^
	158.0912	[Arg + OH − NH]^+^
	175.1195	[Arg-OH + 2H]^+^
	304.1664	[M + OH + 2H]^+^

## Supplementary Materials

The following are available online at https://www.mdpi.com/article/10.3390/toxins13110813/s1, Figure S1: The standard curve of NOD quantified by HPLC (0.01–2.00 mg/L), Figure S2: Five substance peaks in the TIC during degradation. (a) NOD; (b) Linear NOD; (c) Tetrapeptide; (d) Tripeptide; (e) Dipeptide, Figure S3: UPLC-MS/MS profiles for NOD and linear NOD. (a) MS spectrum for NOD; (b) MS/MS spectrum for NOD; (c) MS spectrum for linear NOD; (d) MS/MS spectrum for linear NOD, Figure S4: UPLC-MS/MS profiles for degradation products. (a) MS spectrum for tetrapeptide; (b) MS/MS spectrum for tetrapeptide; (c) MS spectrum for tripeptide; (d) MS/MS spectrum for tripeptide, Figure S5: UPLC-MS/MS profile of dipeptide. (a) MS spectrum of dipeptide; (b) MS/MS spectrum of dipeptide, Figure S6: The dynamics of peak intensity of NOD and degradation products. (a) NOD; (b) linear NOD; (c) H-Glu-Mdhb-MeAsp-Arg-OH; (d) H-Mdhb-MeAsp-Arg-OH; (e) H-MeAsp-Arg-OH.
