# Supplementary material for: Biodegradation of Nodularin by a Microcystin-Degrading Bacterium: Performance, Degradation Pathway, and Potential Application
Source: Toxins (Basel). 2021 Nov 18;13(11):813. doi: 10.3390/toxins13110813 (PMC8618024; doi:10.3390/toxins13110813)
Supplement: Supplementary file 1 [file toxins-13-00813-s001.zip › toxins-1451208-supplementary.pdf]

Supplementary Materials: Biodegradation of Nodularin by a Microcystin-Degrading Bacterium: Performance, Degradation Pathway, and Potential Application

Mengxuan Yuan, Qin Ding, Rongli Sun, Juan Zhang, Lihong Yin and Yuepu Pu

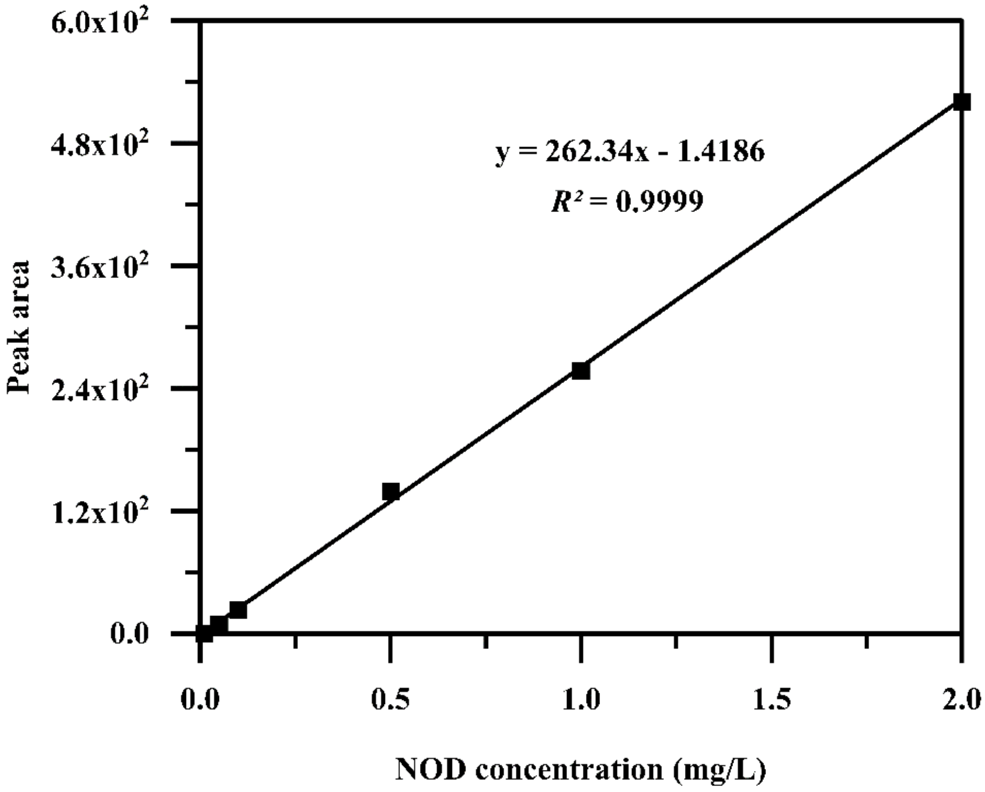

Figure S1. The standard curve of NOD quantified by HPLC (0.01–2.00 mg/L).

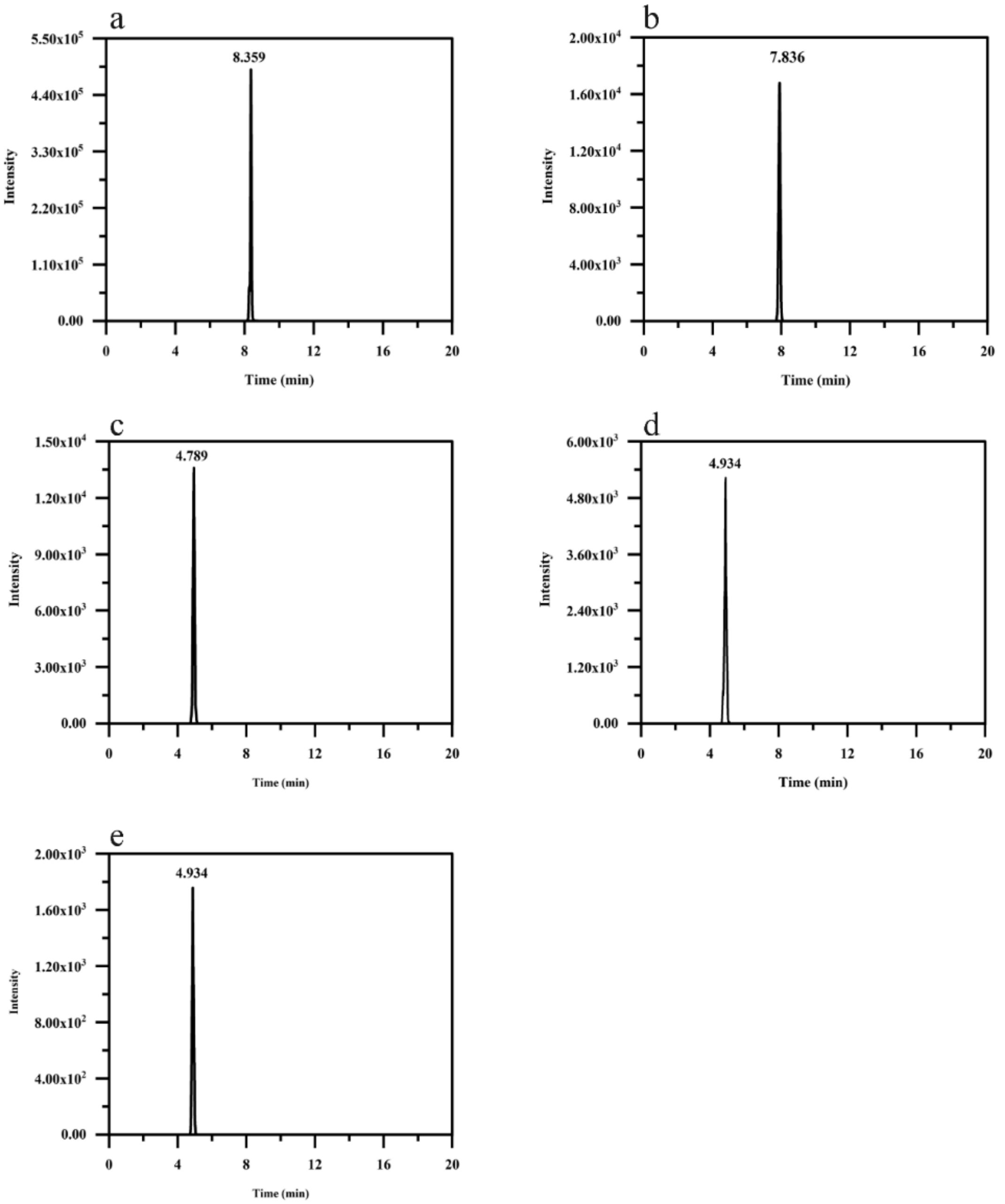

Figure S2. Five substance peaks in the TIC during degradation. (a) NOD; (b) Linear NOD; (c) Tetrapeptide; (d) Tripeptide; (e) Dipeptide.

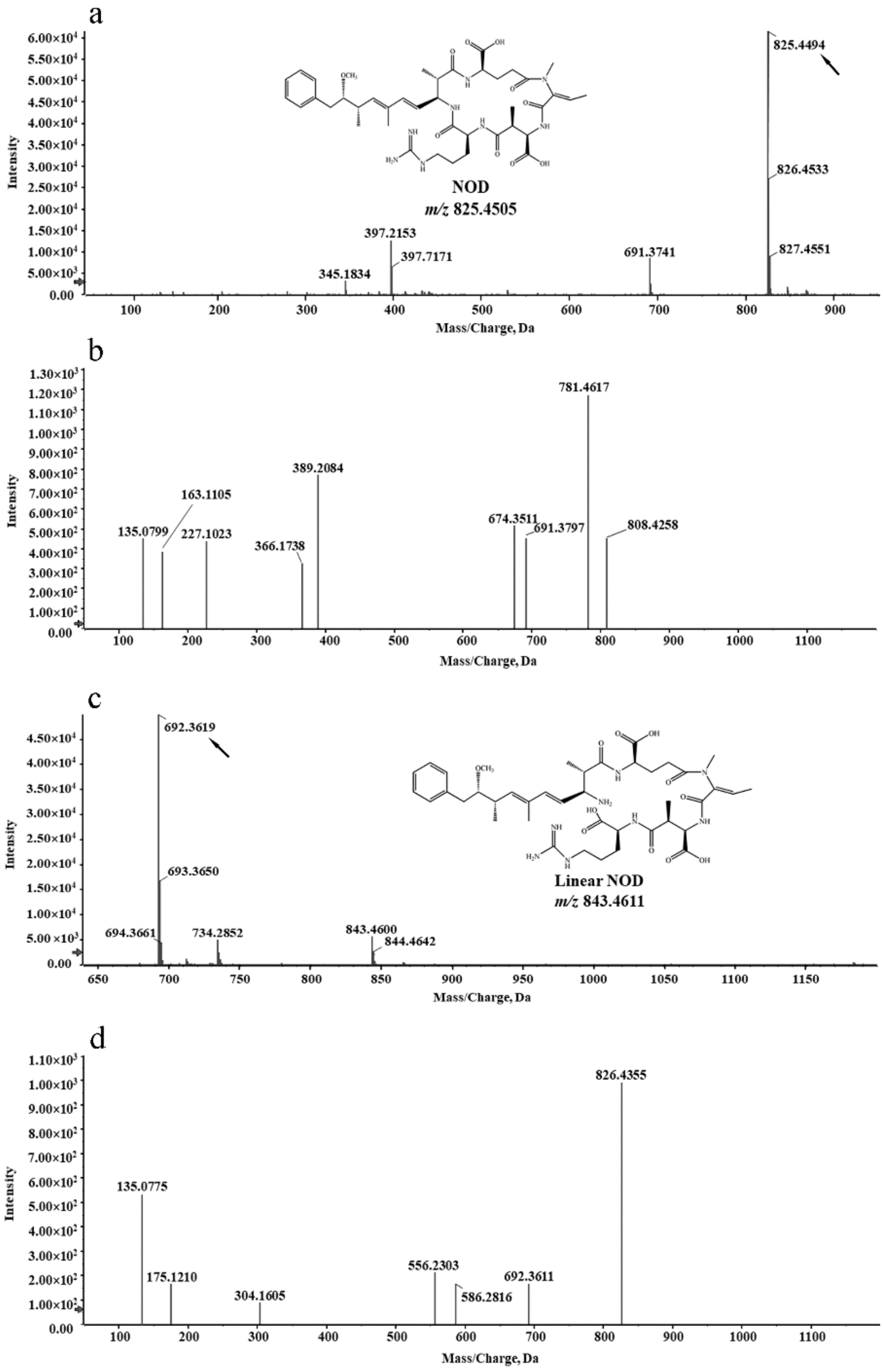

Figure S3. UPLC-MS/MS profiles for NOD and linear NOD. (a) MS spectrum for NOD; (b) MS/MS spectrum for NOD; (c) MS spectrum for linear NOD; (d) MS/MS spectrum for linear NOD.

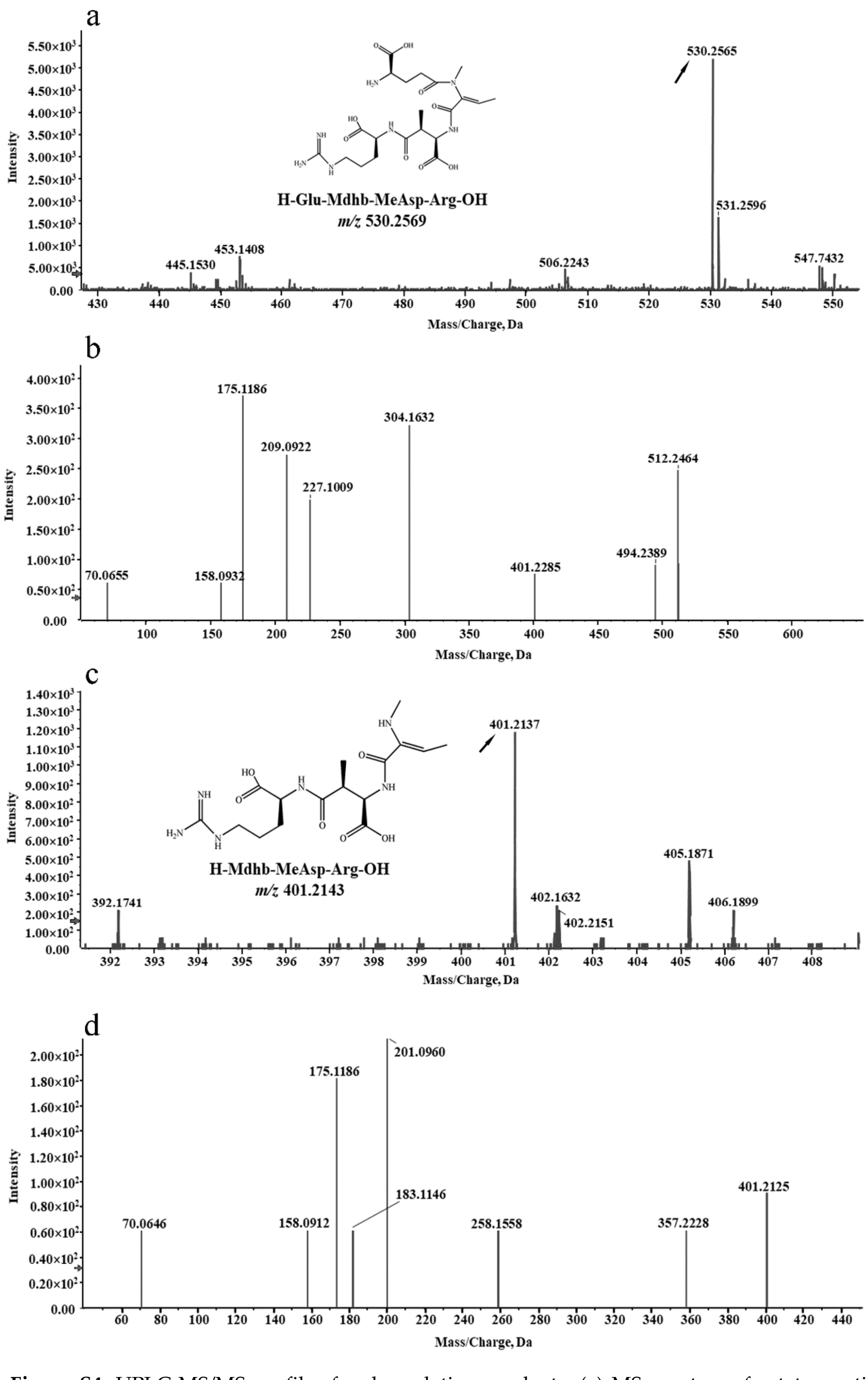

Figure S4. UPLC-MS/MS profiles for degradation products. (a) MS spectrum for tetrapeptide; (b) MS/MS spectrum for tetrapeptide; (c) MS spectrum for tripeptide; (d) MS/MS spectrum for tripeptide.

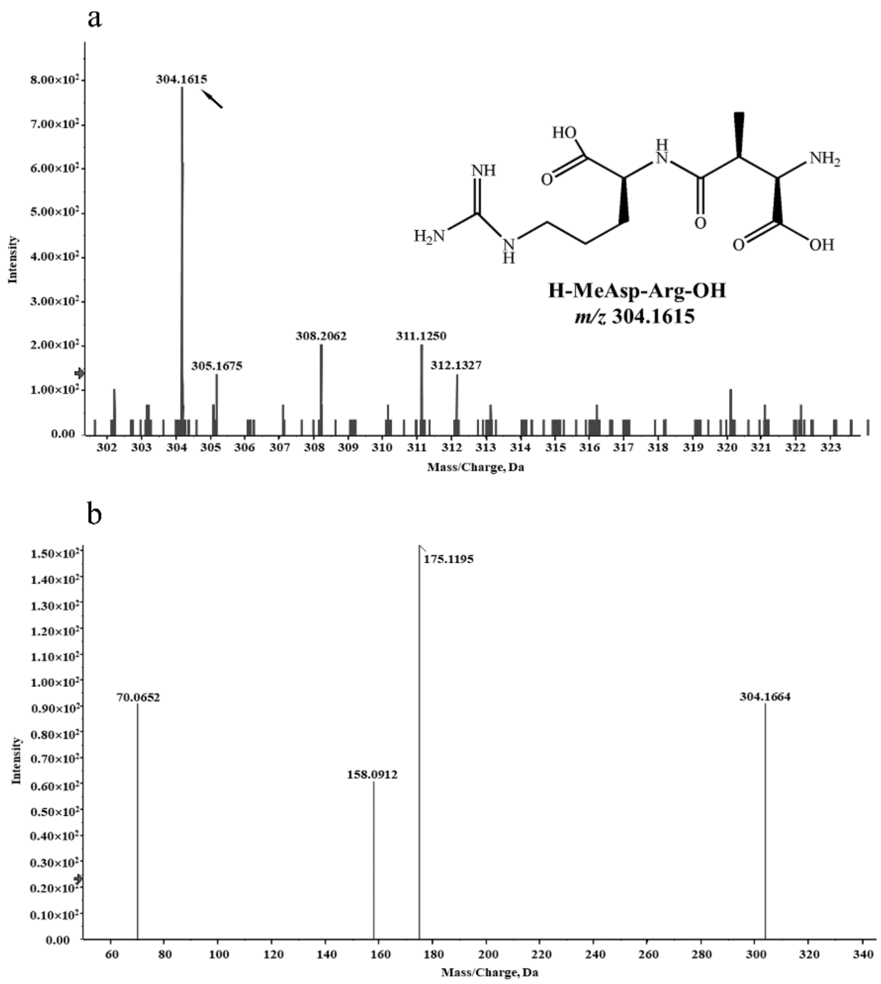

Figure S5. UPLC-MS/MS profile of dipeptide. (a) MS spectrum of dipeptide; (b) MS/MS spectrum of dipeptide.

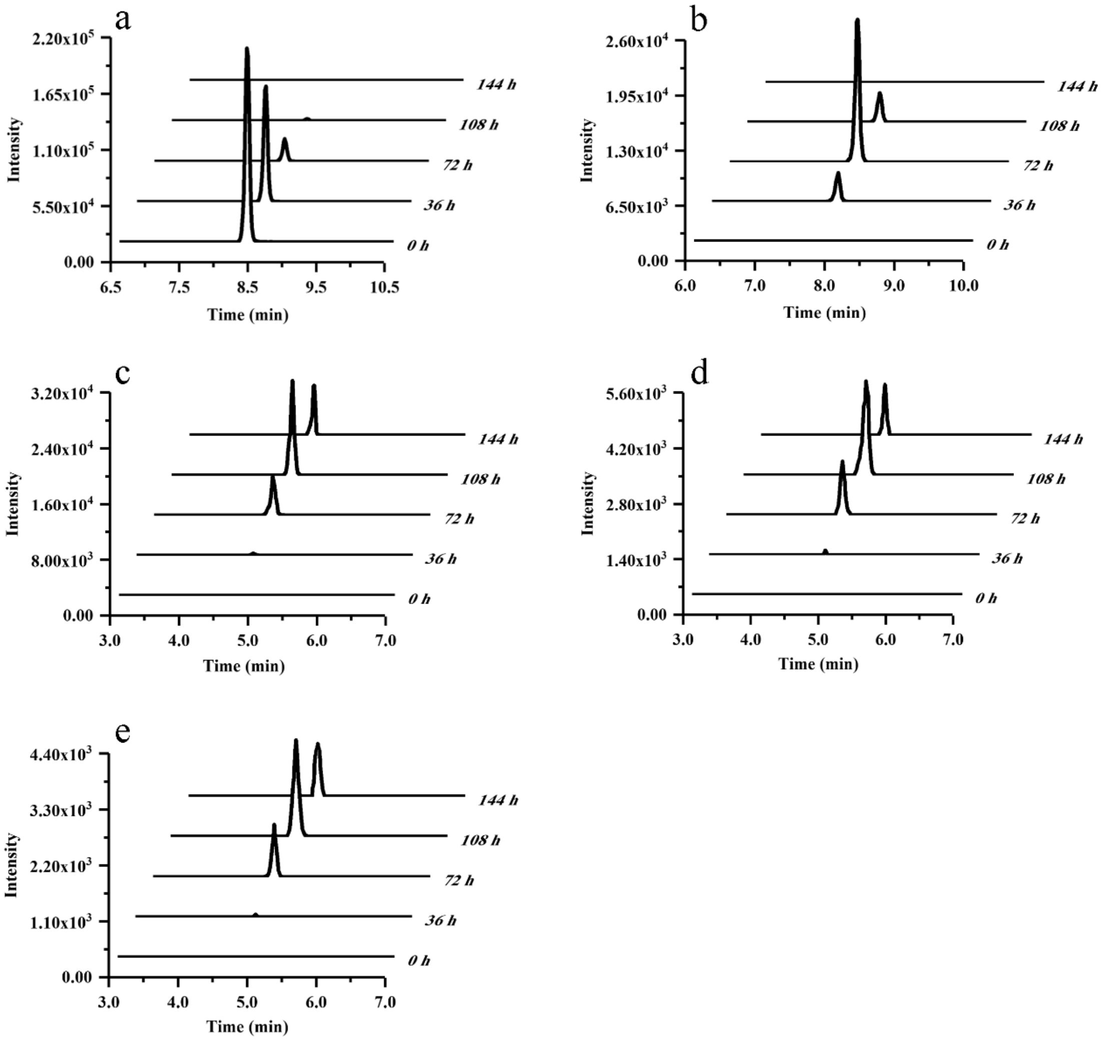

Figure S6. The dynamics of peak intensity of NOD and degradation products. (a) NOD; (b) linear NOD; (c) H-Glu-Mdhb-MeAsp-Arg-OH; (d) H-Mdhb-MeAsp-Arg-OH; (e) H-MeAsp-Arg-OH.
